# Scaling-up production of plant endophytes in bioreactors: concepts, challenges and perspectives

**DOI:** 10.1186/s40643-021-00417-y

**Published:** 2021-07-15

**Authors:** Seedhabadee Ganeshan, Seon Hwa Kim, Vladimir Vujanovic

**Affiliations:** Department of Food and Bioproduct Sciences, University of Saskatchewan, 51 Campus Drive, Saskatoon, SK S7N 5A8 Canada

**Keywords:** Plant endophytes, Scale-up fermentation, Bioreactors, Process development optimization

## Abstract

The benefit of microorganisms to humans, animals, insects and plants is increasingly recognized, with intensified microbial endophytes research indicative of this realization. In the agriculture industry, the benefits are tremendous to move towards sustainable crop production and minimize or circumvent the use of chemical fertilizers and pesticides. The research leading to the identification of potential plant endophytes is long and arduous and for many researchers the challenge is ultimately in scale-up production. While many of the larger agriculture and food industries have their own scale-up and manufacturing facilities, for many in academia and start-up companies the next steps towards production have been a stumbling block due to lack of information and understanding of the processes involved in scale-up fermentation. This review provides an overview of the fermentation process from shake flask cultures to scale-up and the manufacturing steps involved such as process development optimization (PDO), process hazard analysis (PHA), pre-, in- and post-production (PIP) challenges and finally the preparation of a technology transfer package (TTP) to transition the PDO to manufacturing. The focus is on submerged liquid fermentation (SLF) and plant endophytes production by providing original examples of fungal and bacterial endophytes, plant growth promoting *Penicillium* sp. and *Streptomyces* sp. bioinoculants, respectively. We also discuss the concepts, challenges and future perspectives of the scale-up microbial endophyte process technology based on the industrial and biosafety research platform for advancing a massive production of next-generation biologicals in bioreactors.

## Introduction

The pioneering research leading to the discovery of beneficial group of microorganisms is a long and arduous process, often a culmination of over decades’ long work. Recent studies have found that endophytic microbes (mostly fungi and bacteria) residing asymptomatically within hosts can influence human and animal health (Berg et al. 2020), as well as plant fitness (Turner et al. 2013; Rana et al. 2020) including crop yield (Chitnis et al. 2020). In times of modern microbiology, the increasing global market for microbial discovery and identification creates ~ $1 trillion potential (Behera and Varma 2017), while diversification of microbial products using large-scale fermentation in bioreactors (Pham et al. 2019) appears convenient for supplying the beneficial plant bioinoculants to agriculture industry (Mitter et al. 2021); the world largest manufacturing sector at the heart of the global food system (FAO 2017). Indeed, global demand for endophytic bioinoculants has received increasing attention, gaining prominence and market scale in agriculture. Nowadays, this industry is more receptive to the use of high-quality strains in the form of biofertilizers and biopesticides. However, the major challenge in biotechnological innovation remains scale-up fermentation and low-cost products to mitigate food insecurity and agriculture sustainability issues under climate change (Santos et al. 2019). The scale-up production of plant endophytes is still not well documented (reviewed in Parnell et al. 2016), yet the advent of microbial biotechnology has helped in establishing the facts that endophytic microbes have recognized multifunctional prospects and play important roles in industry such as agriculture, food processing, pharmacy, and medicine (Fadiji and Babalola 2020). Noteworthy, the use of microbial endophytes in agriculture to plant growth promotion, resilience improvement to multiple abiotic and biotic stressors, and clean up of environmental pollutants has gained momentum in past years (reviewed in Berg et al. 2015, 2020). The plant endophytes-based innovation for improved yield is a much needed catalyst in agriculture considering that by 2050 the world population is estimated to reach over 9 billion with a necessity to double the food production (FAO 2017, 2018). The latter has to be achieved out of the 13.2 billion ha global land area, about 12% of which is used for agricultural crops (FAO 2011). The agriculture research and innovation clusters are making remarkable progress in isolation, identification and characterization of such plant beneficial microbes, and their testing on crops under controlled environment chamber (phytotron), greenhouse and field conditions. However, the microbial biotechnology science has to surmount actual challenges for efficient fermentation, scale-up production, shelf-life, and formulation for thousands of newly characterized isolates/strains for creation of next-generation biologicals (Murphy et al. 2018; Santos et al. 2019). The success of the endophytic microorganisms in the agri-food system is largely dependent on capabilities to manage an increasing amount of complex data and develop a set of computational information system on specific plant–microbe and microbe–microbe interactions at the cellular, molecular and metabolic levels. The end-result is the implementation of a well structured and organized database for the newly acquired knowledge in the creation of precision agriculture technologies based on promising microbial endophytes with beneficial/desirable function(s).

**Fig. 1:**
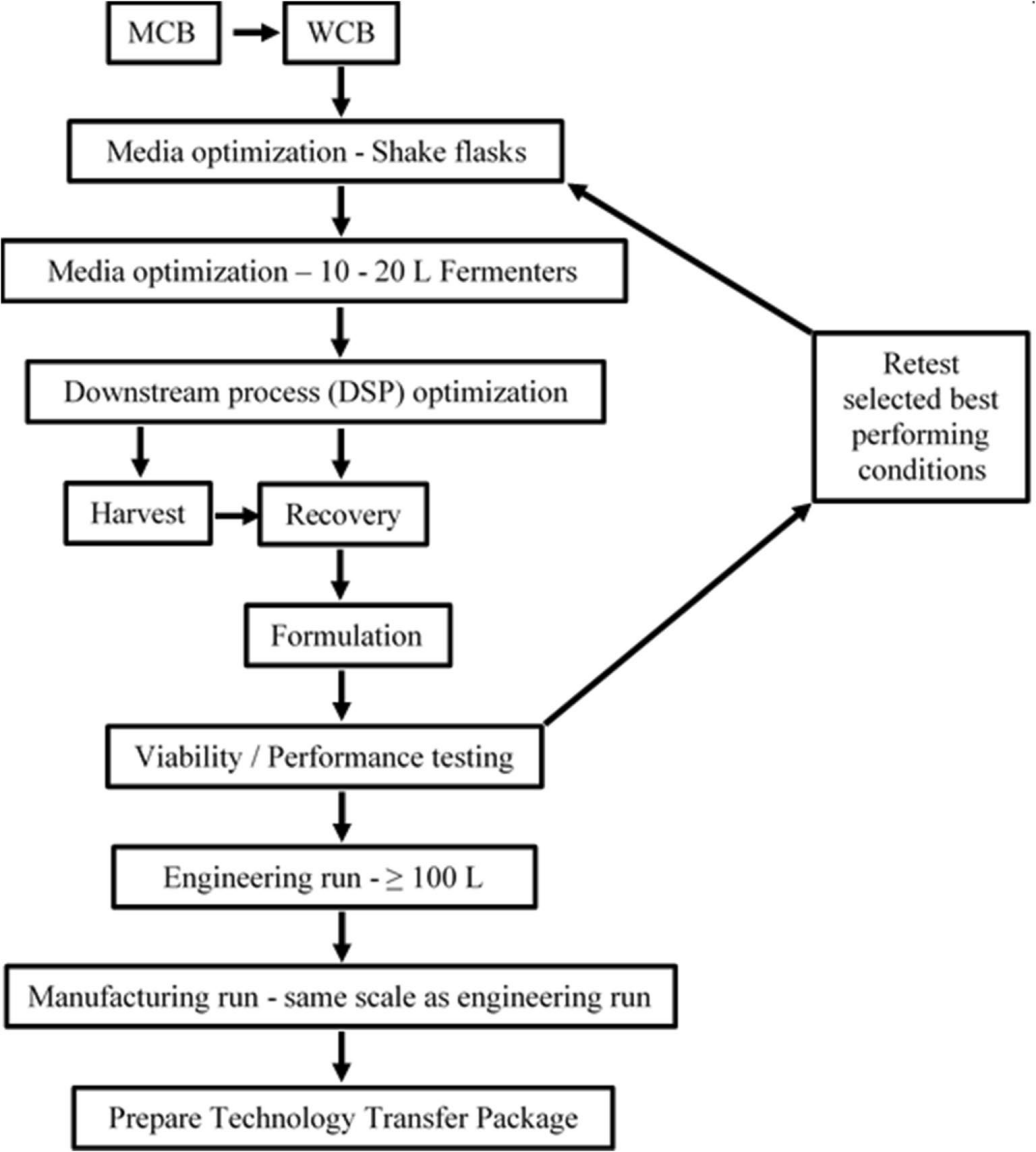
A generalized schematic for fermentation scale-up process

Once the beneficial roles in crop production enhancements of the newly discovered endophytic microorganisms have been ascertained, the focus shifts to process development optimization and large scale-up production. In this review we focus on the scale-up challenges per se from the point of view transitioning the work done in the laboratory to a robust production, i.e. Process Development Optimization (PDO) (Fig. 1), based on our experience and perspectives for plant endophytes. This review is designed to help researchers who have conducted the ground-breaking work to identify and characterize beneficial endophytic (plant growth promoting and/or biological control agents) microorganism(s) of interest and are one step closer to scaling-up production. We focus on submerged liquid fermentation (SLF) as opposed to solid state fermentation (SSF) which would be out of scope in this review. Suffice to say that SSF is the process of growing microorganisms with minimal water or moisture content in the presence of solid substrates (Soccol et al. 2017). Considerations, expectations and information needed in the form of a Technology Transfer Package (TTP) are discussed to facilitate the transition for PDO and scale-up through a contract research organization (CRO) or contract manufacturing organization (CMO). Some examples based on our own studies are also presented to highlight a few of the major practical considerations when scaling-up production.

## Scale-up production challenges

Despite the fact that several technological challenges have been resolved for most of the industrial microbes such as recombinant bacteria and yeasts (reviewed in Junker 2004), our knowledge-based industrial process or technology of bioreactor design phases for efficient scale-up production of plant endophytes is still underdeveloped. The biological questions often get overlooked or not given enough considerations for special requirements of endophytes due to their intracellular lifestyles and growth within living (asymptomatic) healthy plant tissue (reviewed in Reinhold-Hurek and Hurek 2011). Besides the questions that revolve around scale-up challenges in the production process development optimization, encompassing upstream as well as downstream elements of the processes (Crater and Lievense 2018), there is also a need for being reminded that one is dealing with a biological entity, wherein biological cell properties may fluctuate during the adaptation process to new fermentation environment, no matter how much control systems are put in place. Therefore, in addition to bioprocess design aspects, one needs to assess mitigation of microbial endophyte cell growth requirements and its adaptive shift in thermo-dynamic, physico-chemical and molecular traits, as the endophyte should grow outside plant host cells and inside the bioreactor during each particular steps of scale-up fermentation.

The translation of research from the laboratory scale to a manufacturing process (pilot) to large-scale commercial production deserves a lot of considerations in terms of logistics, financial resources, timelines and end goals (Crater and Lievense 2018). There are different ways of assessing the challenges to scale-up, but one of the best strategies would be to take a Process Hazards Analysis (PHA) approach at every step of the scale-up process. The scale-up process itself can be broken down into pre-, in- and post-production (PIP) phases, each with its own sets of challenges. The PHA can be applied within each of the PIP phases to mitigate risks to the scale-up process. However, prior to addressing the PIP challenges and PHA in scale-up production, there is still a gap to be bridged between research conducted in the laboratory and process development optimization (PDO). The PDO is often fast-tracked for the sake of time savings in production and often the lines between PDO and the process leading to production are blurred. The consequences of these expedient approaches are sub-optimal processes with poor yield, poor product performance and the likelihood of significant economic losses in the long term. It is therefore imperative to properly design the optimization efforts from the shake flasks research stage with a view to scaling-up production in a fermenter.

## Scale-up production concepts

The generalized process flow chart gives an overview of the major steps involved in the scale-up process (Fig. 1). Establishment of the Master Cell Banks (MCB) and Working Cell Banks (WCB) are important to ascertain that the integrity and purity of the strain of interest are not questionable during later phases of the process development and/or manufacturing. It is a good laboratory practice to establish an MCB early in the research phase, even during shake flask optimization stages. The MCB once established should be validated for the strain’s identity through molecular approaches and also assessed for purity. Once validated the MCB is used to create the WCB, which also needs to be validated. The MCB serves as the primary stock that is only used to create the WCB stocks to ensure a long-term supply of the strain is available (Chartrain and Chu 2008). The WCB is used for all routine PDO and manufacturing purposes. It is therefore important to make a large stock of the MCB and WCB. Contaminating microorganisms present in the MCB would be carried over to the WCB and proliferate rapidly and overtake strain of interest during scale-up fermentation. One of the typical laboratory environmental contaminant, *Bacillus subtilis*, is known to be such an opportunistic contaminant of cryo-stocks (Stanbury et al. 1995). Given the rich media and ideal conditions in a fermenter, the *B. subtilis* antagonist rapidly overtakes microorganisms of interest, which therefore leads to a failed fermentation incurring loss time and financial resources.

## Laboratory-scale shake flask cultures of plant endophytes, optimization parameters and limitations

Most of the research in the laboratory during the characterization phase of the microorganism is conducted on solidified medium or in liquid medium. The solidified media are generally in the form of agar plates with culture components of interest for colony isolation, colony forming units (CFU) counts, colony morphology and characteristics assessments, etc., as standard practices (Nicolaidou et al. 2019) generally taught to bioprocess scientists, students, researchers, biosafety officers, company managers and industry professionals. The liquid cultures, on the other hand, are conducted to generate material for bioassays, metabolites detection and quantification, growth chamber/greenhouse tests for efficacy assessments and biomass yield determination. While media components would have been judiciously selected for optimal biomass yield, such optimized media may not always translate to similar efficiencies when used in fermenters due to more sophisticated control parameters in the latter compared shake flasks. Furthermore, cost considerations for media components may not have been given due considerations at the shake flask stages when growing 1–2 L of culture. Expensive media components may be suitable for shake flask cultures, but at larger volumes, as in fermenters ranging in volumes from 100 to 1000 L or more, such expensive components would rapidly add to the cost of production. Thus, prior to starting PDO it is important to develop a sound strategy for media optimization in shake flasks, with the understanding that further modifications to the media may be necessary during PDO in the small-scale fermenters. Those modifications are generally less elaborate and involve comparing the quantitative nature of media components in concurrent or back-to-back fermentation runs rather than the specific media components. In our own studies in shake flasks for a fungal strain of an endophytic biocontrol agent, we have compared six different media and tested the best two media based on wet/dry cell weights in 15 L fermenters.

Shake cultures, whether in flasks or bottles, have been in existence for over 85 years and during that period various monitoring approaches have been devised to measure and improve growth (reviewed in Takahashi and Aoyagi 2018). Although regulation of parameters such as temperature and agitation have progressed with design and engineering of incubator-shakers, other factors such as aeration (for oxygen supply) and pH have been more challenging to control. In aerobic cultures, oxygen availability throughout culture is critical to maintain cell growth. The initial oxygen present in the culture broth rapidly becomes limiting as cells growth and the agitation allows oxygen exchange to take place. The agitation provided by incubator-shakers allowed atmospheric oxygen supply by increasing the air–liquid contact surface area to maximize oxygen transfer rates (OTRs). To further increase the air–liquid surface area or OTR, other developments in flasks such as baffles were designed (for review see Takahashi and Aoyagi 2018). Baffles have been shown to increase OTRs 6–12 fold at various agitation speeds (Running and Bansal 2016). Although it may appear that higher agitation and more baffles would significantly increase OTR in the culture broth, this may not be the case, since shear forces with these physical and mechanical modifications would increase as well and cause physical damage to the cells. With bacterial plant endophytes, we have not observed any adverse effects in growth due to OTR limitation even up to 4 L working volume in a 5 L baffled shake flask. However, with fungal plant endophytes we observed growth inhibition for some strains after an initial vigorous growth. This initial growth led to rapid depletion of nutrients, but resulted in large amounts of biomass at the 4 L working volume.

The working volume for the cultures will also be limited at higher agitation due to potential for splashing. The splashing with a small working volume in a large flask may not be of concern compared to a larger working volume in a similar flask. Splashing within the flasks under these conditions could compromise sterility if flask plugs were exposed to the broth and in turn the broth being siphoned to the external environment. The working volume to flask volume ratio is generally kept at 1:9 to 4:6, depending on the geometry of the flask. Fernbach flasks, for example, have a large surface area to volume ratio (wider base compared to the height of the flask) and therefore a larger volume could be used without the risk of splashing. Typically for a 2.5 L Fernbach flask, up to 1 L of culture broth can be used at an agitation of 250 rpm, with minimal risk of splashing. However, caution should be exercised as to the speed due to potential for foaming of the broth at high speed. Foaming is dependent on the constituents of the broth and the higher the speed the more foaming is likely to occur. It is therefore important to observe for foaming at the onset of the experiment and adjust the agitation speed accordingly. Foaming can potentially lead to contamination if allowed to reach the mouth of the flask and pushed to the outside. Uniform temperature maintenance within culture flasks is also challenging with increasing working volume. Temperature distribution within a flask at 100 mL working volume will be more uniform than a flask with 1000 mL or more working volume, in spite of increased agitation. The latter is of course subject to the above-mentioned consequences of splashing.

The control of pH in shake flask cultures is another challenge. Depending on the microorganisms being cultured, there is always the potential for pH shifts due to depletion of nutritional components in the broth as well as production of metabolites during growth. The pH shift to acidic or alkaline range can profoundly affect growth of the microorganisms, prevent production of beneficial metabolites or lead to accumulation of toxic metabolites (Weuster-Botz et al. 2001). Automated controls of pH in shake flasks have been given considerations and various attempts have been made. The challenge, however, has been with online monitoring and adequate pump systems to control changes in pH in shake flasks and potential sensor systems and control systems have been attempted (Ude et al. 2015). Notwithstanding the ongoing search for automated monitoring and control systems for shake flasks, limitations still remain for the scale-up of production, especially when it comes to the maximum working volumes which can be achieved in shake flasks. Similar to OTR limitations, pH drift also affects growth of microorganisms. In our studies, we have not observed significant growth inhibition in shake flask cultures of fungal plant endophytes. Generally, the initial pH for our fungal strains has been 5.0, and drifts to 4.0 or 7.0 did not affect growth. With bacterial plant endophytes, pH of culture medium is usually adjusted to 7.0 and we have not observed significant pH drifts in shake flask cultures.

## Versatility of fermenters for plant endophytic microbial growth over shake flasks

As mentioned above, laboratory-shake flasks, while useful for early phase of research, become limiting when transitioning to scale-up production. Fermenters or bioreactors have reached very high levels of sophistication in terms of engineering design and controls to overcome the limitations encountered in a shake flask process. Computers and biotechnology software are allowing highly accurate assessment and data analytics (Kakes 2008) in helping to develop consistent and reliable operating protocols for all stages of development, scale-up production and manufacturing. Since new technology comes with an increased level of sophistication, it also comes with higher demand for enhanced efficacy, yield and quality of products, and reduced operational costs. Therefore, careful defining of the objectives, proper planning and judicious design of experiments are required to mitigate preventable failures in fermentation and avoid cost overruns (Reisman 1988). One of the foremost consideration at the outset would be the medium composition optimization as this would dictate cost of production and propensity for product separation from broth in downstream processes with little interference on the final quality of biomass produced (Kennedy and Krouse 1999). It is also about the relevance of the use of specific waste substrates adapted to endophytes that may contribute to reducing the costs of commercial production (Elsallamet et al. 2021; Robl et al. 2020; Willaert 2021). Such a solution may mark future of the green scale-up fermentation and sustainable development strategy.

**Fig. 2:**
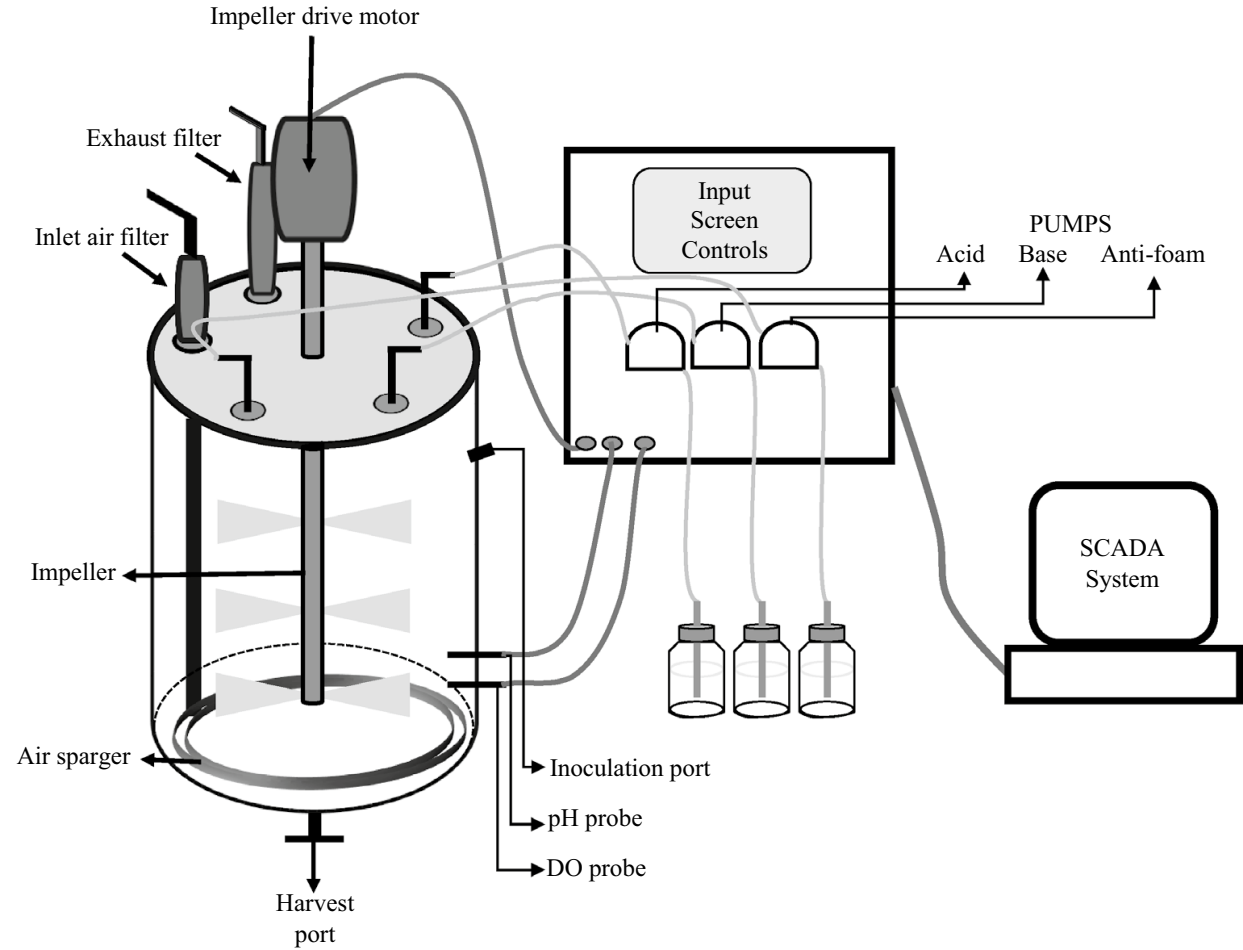
Schematic of a generalized stainless-steel fermenter of 15–200 L scale, associated controls and accessories

While a number of fermenter designs have been described (for review see Matthews 2008), this review focuses rather on certain features delineating the benchtop or industrial scale bioreactors from shake flasks and small-scale laboratory fermenters. Small-scale fermenters can be considered to be in the range of 1–20 L volumes (Matthews 2008) and these are ideal for media testing and optimization. Among these ranges, generally the 1–10 L fermenters are glass designed and autoclavable. While adequate in terms of volume, they are prone to damage, requiring careful handling. Fermenters of 15–30 L volumes are better suited for process development as they are clean-in-place (CIP) and sterilize-in-place (SIP). Besides the subtle practical aspects of handling between the glass fermenters and the stainless-steel fermenters, the most important features of fermenters over shake flasks are the physical designs control systems (Fig. 2). The fermenters are closed systems capable of maintaining sterility for long periods, provided external inputs feeding into them are leak-proof. Among the control systems in fermenters which bring the most desirable advancements over shake flasks are those for pH, temperature, aeration and agitation. Another advantage with fermenters over shake flasks is with the ability to remove sterile samples for various analyses such as optical density measurements (OD), plating, metabolites assays, etc., during culture.

The pH is monitored by a pH probe connected to a display. pH fluctuations are provided in real-time and peristaltic pumps attached to the fermenter allow automated addition of acid or base to the fermenter via sterile tubing, should the pH deviate from the set-point. Similarly, dissolved oxygen (DO) in the broth is monitored by a DO probe, again connected to the monitoring device. Starting DO for any fermentation is 100% and as the microorganism grows, oxygen is consumed and the DO level slowly declines. The minimum DO level can be set (e.g., 30 or 40%) and once the minimum set-point level is reached, two control systems can be set for increasing DO levels in the culture medium. One is the increase in aeration cascade. The aeration is generally set as vessel volume per minute (vvm) or litres per minute (lpm) at the onset of fermentation at a certain level in vvm or lpm units. One vvm air in the headspace of the fermenter is equivalent to one volume broth in the vessel. Thus, if the fermenter working volume is 10 L, the starting vvm can initially be set as 0.5 vvm. In terms of lpm units, this will be equivalent to the volume of broth in the fermenter, i.e. 5 lpm. The aeration cascade can be set such that when the DO drops to the minimum set-point, the aeration increases to the maximum of 1 vvm by the automated sterile air inlet airflow controller which allows more sterile air into the fermenter headspace. The consequence of this would be an increase in DO in the broth.

However, when the aeration has reached the maximum set-point and the DO cannot be maintained at the minimum set-point, then the agitation cascade can be set to start. The starting agitation, provided by the impellers, is generally set at 100–200 rpm. When the DO becomes limiting, the agitation cascade can be set to increase to a certain maximum (e.g., 500 rpm). This is determined based on fermenter mechanical design according to the manufacturer recommendations. The increased agitation allows better dispersion of oxygen in the medium leading to an increase in OTR. The role of the impellers besides aeration is also to allow mixing of media and biomass in the fermenter, thus allowing maximum contact of cells with the nutrients in the broth. Impellers also allow even distribution of the temperature throughout the fermenter, especially critical at larger volumes. Thus, with just the few controls mentioned above, all of the limitations associated with shake flasks can be overcome.

## Control and data acquisition for microbial endophytes in scale-up production system

**Fig. 3:**
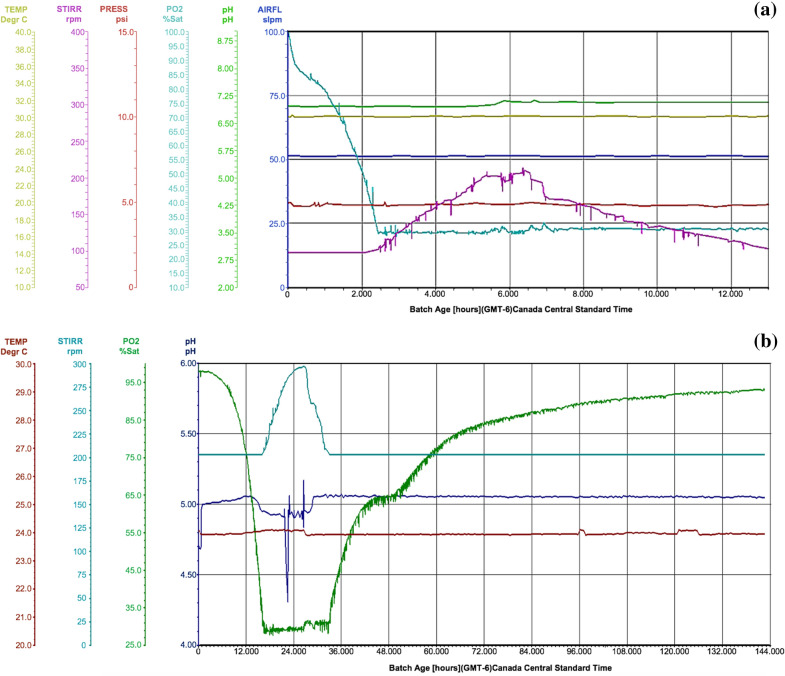
Typical SCADA plot generated during a scale-up fermentation run for a **a** bacterial endophyte *Streptomyces* sp. SMCD strain, **b** a fungal plant endophyte *Penicillium* sp. SMCD strain

In terms of the scale-up production, fermenters are generally designed for monitoring and real-time collection of data through various interfaces to a Supervisory Control and Data Acquisition (SCADA) system and software (reviewed in Kakes 2008). A typical SCADA plot would consist of some basic run information parameters that a researcher would want to collect to assess the efficiency of the run (Fig. 3) and depending on requirements, more advanced data collection can be performed as well. For example, the above-mentioned controlled parameters such as pH, temperature, DO and agitation can all be plotted in real-time allowing immediate assessment of the run (Fig. 3). This becomes more relevant for overnight fermentation runs, when it is not always practical or critical to have personnel on site as when fungal endophytes are being grown. As an example, among the bacterial and fungal endophytes used for improved crop resilience to drought and heat (Vujanovic and Germida 2017), *Streptomyces* sp. SMCD strain (Kumari et al. 2018; Vujanovic and Germida 2014) grown in a 30 L fermenter showed a rapid decline in DO within the first 2 h of fermentation, indicating vigorous growth as reflected in the SCADA plot (Fig. 3a). As the DO reached the minimum set-point of 30%, the agitation ramped up to meet oxygen demand. Such post hoc observations of the plot allow assessment of the run and underlie the importance of adequately installed SCADA systems. Fermentation was terminated after 12 h since no further growth was observed. Compared to bacterial endophytes fermentation of which can be completed it 1–2 days, fungal endophytes are generally slower growing, requiring extended duration (5–7 days) of fermentation. A quick assessment of SCADA plot from such overnight runs would indicate if any of the automated control systems failed or if the minimum DO could not be maintained because the agitation and aeration cascades had reached the maximum set-points. Fermentation of the fungal endophyte *Penicillium* sp. SMCD strain (Vujanovic et al. 2019), for example, lasted 6 days (Fig. 3b). While the run proceeded smoothly, it can be observed from the SCADA plot (Fig. 3b) that between 22 and 30 h there was some fluctuation in the pH. This was due to a defective alkali addition pump, which therefore prevented pH adjustment to the set-point of 5.0. Since the operating staff was onsite during this occurrence, the defective pump was swapped for a new one without jeopardizing the entire run. With the fungal endophyte fermentations, there is some level of deviation which can be tolerated and not affecting the integrity of the run. For example, the inability to maintain minimum DO level may not adversely affect the viability of the cells, although it may slow growth. Bacterial endophytes on the other hand are less forgiving and cell death may occur due to DO limitations. Generally, for bacterial plant endophytes such as *Streptomyces* sp. fermentations due to their short durations, overnight staffing for monitoring is required.

## Scale-up strategies

The process development optimization should be viewed as a critical part of the scale-up process towards production. It is at this stage that most of the media components, yield, efficiency of the process, other production parameters and even downstream process strategies are envisioned, planned, tested and refined (Matthews 2008). A typical PDO will start with assessing media components already tested in shake flasks and strategies for re-testing in fermenters. At this point, costs of media components are also assessed and to determine if cheaper sources would also be viable (Singh et al. 2017). As a preliminary test, shake flask cultures may be set up to test the new cheaper media component alternatives prior to testing in a fermenter, but with the understanding that in a fermenter there will still be modifications to undertake given the additional controls which are likely to affect growth dynamics (Kennedy and Krouse 1999). Strategies for media optimization have also advanced considerably with greater emphasis on experimental designs and statistical analyses. From the old practices of one-factor-at-a-time approach (OFAT), focus has shifted to Design-of-Experiments (DOE) and Response Surface Methodology (RSM) approaches (reviewed in Mandenius and Brundin 2008; Singh et al. 2017). Irrespective of the approach for determining the optimal media components, the choice of the source of the alternative components are based on economics, if possible. For example, components of the media which are used in large quantities would significantly add to the cost of production at larger scales. The PDO phase without any doubt provides the right opportunity to incorporate such media components adjustments. As an example, the carbon and nitrogen quantities in both bacterial and fungal cultures are significant in quantity. The large quantity of any of these components could therefore add to the cost of production, unless a cheaper alternative of lower quality can be substituted without jeopardizing the yield, quality and performance of the product.

To put the media selection strategies during PDO into perspective, depending on the microorganism of interest, an undefined medium as opposed to a defined medium can be selected. Undefined medium consists of complex natural ingredients with ill-defined compounds, while defined medium consists of chemically defined pure compounds of known proportions (Zhang and Greasham 1999). The use of chemically defined media at the research phase is routine and helps to identify nutritional components which would maximize growth in shake flasks. These nutritional components include macroelements and microelements for various cellular metabolic processes that enable the cells to survive, grow and reproduce. Carbon as a nutrient source for energy, for example, is one of the most important components for growth of microorganisms and is generally obtained from glucose in the culture medium. Various commercial sources of pure grade glucose are available and when added as a component of the culture at a given concentration would constitute part of the defined medium along with other components also at specified concentrations. The concentration needed per litre of culture medium in a shake flask may not seem costly, but when the medium volume is scaled up to 20 or 100 L, the cost of the glucose quickly becomes significant. It is, therefore, imperative that at the research stage considerations are given to explore alternative sources of nutrients. As a substitute for glucose in plant fungal endophytes, we have employed potato dextrose broth (PDB) or molasses as a source of carbon. Such substitutions with complex components would qualify as undefined media components. However, PDB is also expensive compared to molasses, but still cheaper than purified glucose. For example, in our fermentation studies PDB was substituted with molasses for the *Penicillium* sp. strain SMCD and allowed prolific growth of mycelia. Nonetheless, other components can still be defined such as Mg, Fe, K, etc. Nitrogen sources can also be defined or undefined. However, in this case the undefined N sources are sometimes more expensive such as peptones and tryptones and the inorganic nitrogen sources from ammonium salts may serve as better substitutes. As a note of caution, it has to be reiterated that all these media components optimization have to be conducted in shake flasks prior to testing in fermenters and only selected components are tested/further optimized in fermenters or bioreactors. At this stage of the PDO, it is important to collect as much information as possible to improve the growth of the microorganisms through an understanding of their physiology and media components interactions and develop a reproducible culture system (Harvey and McNeil 2008).

Prior to testing the media in the fermenters, as part of the PDO, batch records (BRs) need to be available for entering all the media components and other run parameters intended to be programmed for the fermentation run (reviewed in Kakes 2008). A BR is set up for every process leading to the product recovery. Thus, BRs should be available for each of the processes from shake flask to recovery (Fig. 1). A BR is a fillable document containing the steps for setting up the particular process. For a shake flask culture, it will consist of media components and the required amounts per litre. The fillable section will consist of blank spaces for the actual amount (weight or volume) of the respective media components for the desired culture medium volume. The weighed amounts or measured volumes of medium components are entered into the BR and initialed by the technologist preparing the medium. Similarly, the conditions for incubation of the flask such as agitation speed, and temperature are also entered. Any deviation from the set parameters in the BRs need approval from the supervisor and details are recorded and initialed by the technologist. At the end of the process, the supervisor reviews and approves the BRs, with comments and/or remarks. The BR thus serves as a document for future review and adjustments to processes, while also documenting and validating actual details specific to that particular process conducted. The BRs are given code numbers called batch record numbers which can consist of letters and/or numbers which identify a particular project or client. The BR numbers are equivalent to lot numbers in manufacturing and allow traceability for conducted process. For a fermentation run, the SCADA serves as record of the process data and should be available along with the BR for review. SCADA and BRs are reviewed, approved and archived. During the PDO, the BR serves as the point document from which parameters for future scale-up processes will be based upon and compared for performance, yield and quality of the product.

## Scale-up strategies—PIP

In line with PDO, challenges at the pre-, in- and post-production phases need to be considered and addressed as each of these can severely impact product yield, quality and performance. Indeed, plant endophytes might experience low tolerance to changing environmental parameters in bioreactors (oxygen, pH, pressure, nutrition) compared to optimal conditions determined by each particular microbial environmental niche (root, seed, flower, leaf, soil aggregates, etc.). Compared to microorganisms such as *E. coli* or *B. subtilis* which generally grow well, conditions for plant endophytes have to be judiciously adjusted. This is because these plant endophytes are being cultured out their natural environments within the plant where they were growing as symbionts. Only after ascertaining that vigorous growth on a number of substrates can be achieved should the strain be further advanced (Murphy et al. 2018).

### Pre-production

In this section, some general pre-run considerations and challenges are discussed from an operational perspective. Media optimizations have been discussed above in the context of PDO. This preparation prior to the start of fermentation, referred to here as pre-production, forms the basis for a successful run—importantly preparing and maintaining sterility of the vessels. Since the fermenter is operated as a closed system, with aeration provided from atmospheric air via sterile inlet filters, it is important to ensure that the sterilization process is effective and that there are no inadvertent leaks in the system that may compromise sterility (Matthews 2008). For example, during preparation of the fermenter valves and gaskets in the fermenter have to be examined for wear and tear and defects. As with the integrity of the inlet filters, the exhaust filters need to be checked as well.

As indicated earlier, pH shifts occur in shake flasks and are generally uncontrolled. In fermenters due to higher growth rate and metabolic activity, pH shifts are more significant (Hayward 2008). Therefore, pre-production preparation also includes setting the acid and base addition bottles at appropriate volumes, sterilizing them and aseptically porting them onto the sterile fermenter. The volumes and molarity of the acids and bases will depend on the fermentation scale and the microorganisms. Bacterial fermentations are generally conducted at a pH of about 7.0 and fungal fermentation at pH 5.0. The pH of a terminated shake flask medium can be checked to determine the shift in pH after growth to get an indication if the medium has become acidic or alkaline during growth. Depending on this shift, if large, a 5 M concentration of the acid and base solutions can be prepared. Too low of a molarity will mean more volume being pumped into the fermenter which will lead to a significant increase in volume beyond the working volume allowable by the physical design of the fermenter. Too highly concentrated acid solutions can lead to corrosion of tubing, gaskets and valves, which can compromise sterility of the system. Concentrated acids can also corrode the interior of stainless-steel fermenters.

Addition of antifoam agents will need to be anticipated as well. Foaming in a fermenter occurs due to combination of different factors such as increased air flow and agitation along with specific media components (Matthews 2008). Foaming can pass into the condenser and cause wetting of the exhaust filter, which would prevent exhaust. This can lead to pressure build-up in the vessel. Usually, the foam probe alarm will be triggered in the event of high foam and will lead to, if set up, the addition of antifoam reagent by actuation of the pump. It is to be noted that antifoam addition should be kept to a minimum, as too much of it can affect the growth of cells, DO and downstream processing. An antifoam which is effective for bacterial endophytes fermentation may not be effective for fungal endophytes fermentation. The antifoam can also be added to the fermentation broth prior to sterilization and only added to the vessel during the run manually via a sterile addition bottle set up rather than setting up the automated addition when foam probe is triggered. This would prevent an excessive addition of antifoam to the fermenter.

### In-production

The in-production stage may appear as a fully controlled process which should proceed without any likelihood of failure. However, a number of preventable failures can occur and it is important to closely monitor the run. For example, the pH control can fail due to acid/base pumps malfunction. This would lead to pH drift that can adversely affect growth of microorganisms. In our own experiments (Fig. 3), we have observed that fungal endophytes are less prone to growth interruption as a result of pH drifts over 10–12 h and growth recovers rapidly once pH is readjusted to 5.0. Bacterial endophytes on the other hand are not tolerant to wide pH fluctuations and pH drifts lead to death of cells. This recovery in the growth of fungal endophytes is more likely due to the longer duration of fermentation (5–7 days) compared to bacterial endophytes (24–48 h).

**Fig. 4:**
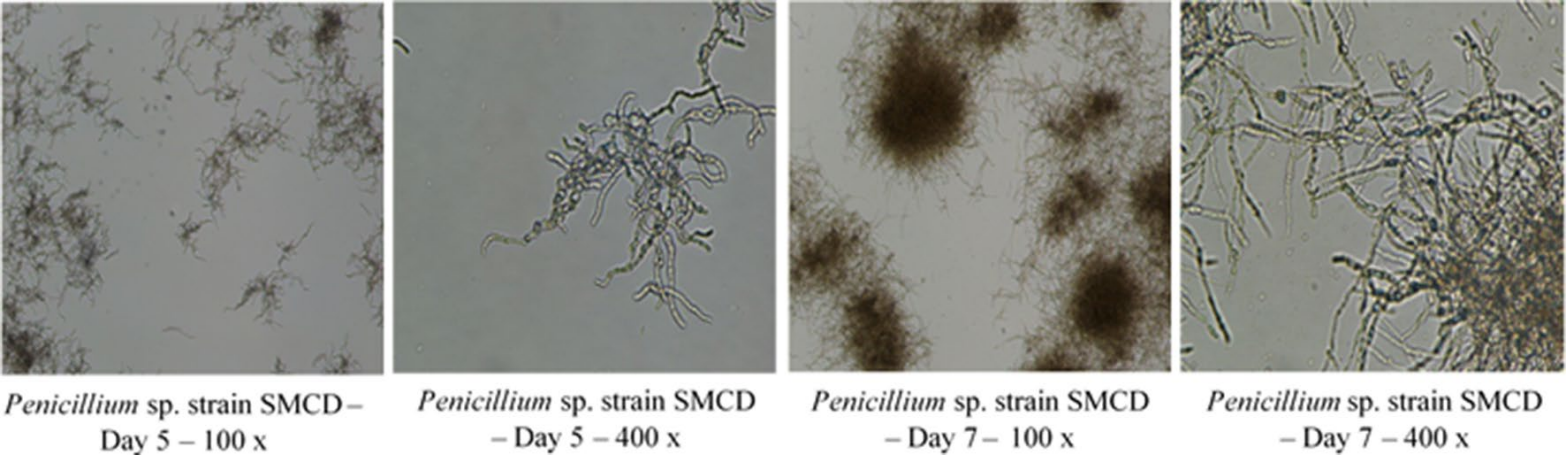
Microscopic observations of scaled up *Penicillium* sp. SMCD biomass production in 300-L fermentation with CFU increasing over growth duration (5–7 days). SMCD strains are from Dr. Vujanovic’s Saskatchewan Microbial Collection and Database

**Fig. 5:**
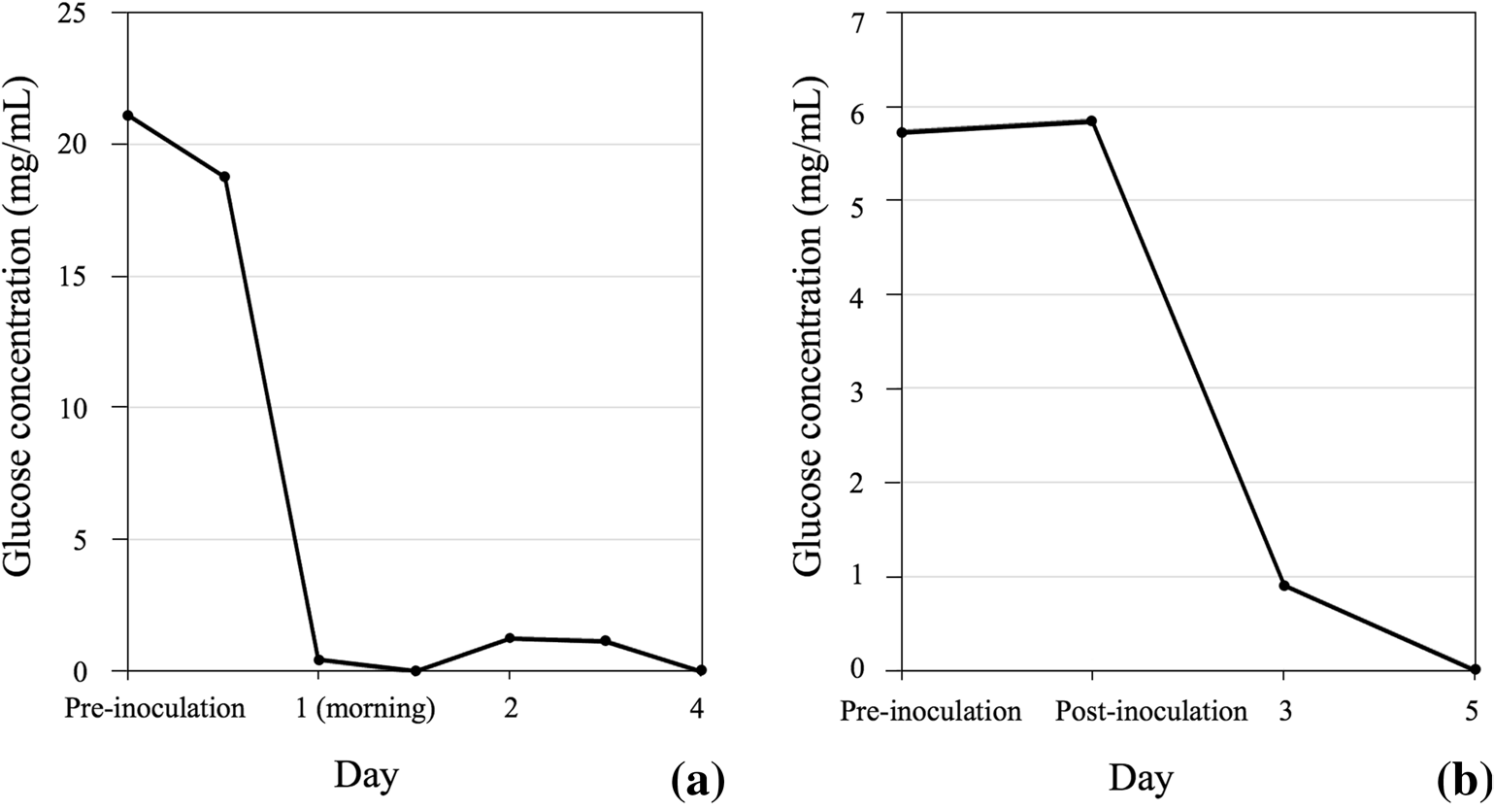
Glucose utilization during scale-up biomass production in 300 L fermentation. **a** *Streptomyces* sp. strain SMCD with glucose depleted by the afternoon of Day 1, with requirement for glucose addition; **b** *Penicillium* sp. SMCD with glucose only depleted after 5 days of fermentation

Critical to PDO during in-production run is in-process testing, which includes all of the criteria needed to assess efficiency of process by way of CFU counts, wet and dry cell weights, optical cell densities (for bacterial cells) and other analytical tests as required. Microscopic observations will ensure a fast assessment of cell morphology and purity (Fig. 4). Assays for media components such as glucose can also be conducted to determine rate of utilization. This will provide an indication as to the growth rate and if glucose would become limiting. If this is the case, a sterile glucose feed can be performed. In our studies with *Streptomyces* sp. SMCD, glucose was depleted by the afternoon of Day 1 (Fig. 5a) and a glucose feed was performed to prolong the growth and increase biomass. With the *Penicillium* sp. SMCD, glucose was only depleted by Day 5 and no glucose feed was necessary (Fig. 5b). CFU and dry cell weight data are available after the run, but valuable for further optimization of the run for efficiency. Importantly, the collection of in-process data during the PDO will enable selection of tests critical to monitor run progress at the manufacturing stage and ascertain performance of the product. This is in-line with quality assurance/quality control (QA/QC) parameters at the manufacturing stage. Depending on the microorganisms, the appropriate QA/QC validation methods would be applied.

### Post-production

**Fig. 6:**
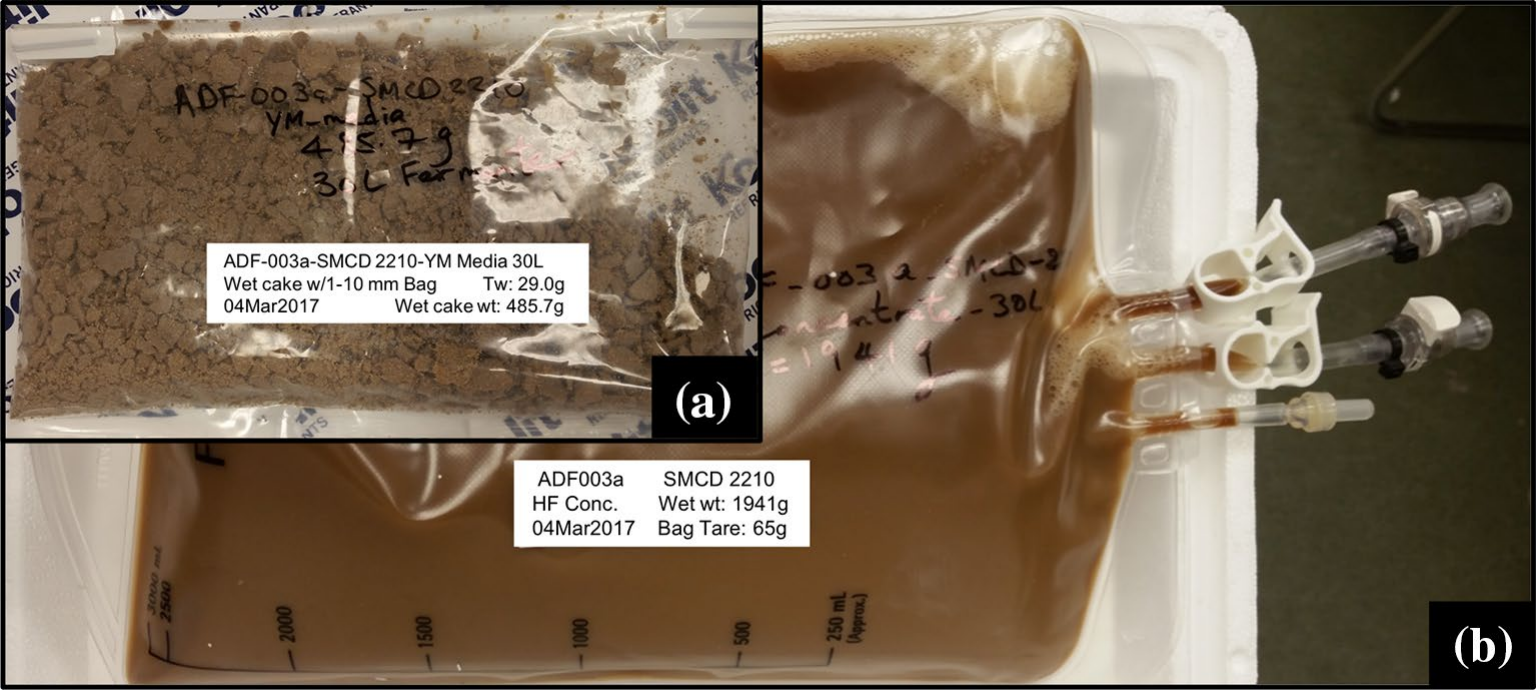
Scale-up *Paraconiothyrium* sp. SMCD fermentation product as **a** wet cake obtained from basket centrifugation and **b** liquid biomass consisting of spores concentrated from the centrate by hollow fibre filtration

Post-production PDO is essentially the downstream processing (DSP) considerations for the fermentation product such as selecting the equipment to use based on microorganisms and the scale of fermentation (Burke 2008; Harvey and McNeil 2008). This would include plans for harvesting, recovering and formulation of the biomass (Fig. 6). DSP for bacterial cells will be different from those of fungal biomass. Bacterial cells can be concentrated by tangential flow filtration (TFF) either through hollow fibre filters or flat sheet membranes. Both filtration systems are available in configurations for small-scale and large-scale processing. For fungal endophytes, a basket centrifuge with a 0.1–1 µm pore size filter bag is ideal for trapping fungal mycelia. The basket centrifuge is usually available at a pilot scale. With some fungal endophytes, spore production can be triggered by selection of media components and/or limiting certain nutrients. We have successfully used approach to trigger spore production in the fungal endophyte *Paraconiothyrium* sp. SMCD (see below). In such cases, the basket centrifugation approach can be used to trap mycelial biomass and the centrate (broth coming out of the centrifuge and containing spores) can be collected and run though TFF to concentrate the spores. High spore production is economical as it allows more effective formulation and treatment covering a larger acreage compared to mycelia (Murphy et al. 2016, 2017).

In our studies, a fungal endophyte strain with plant growth promotion (PGP) and insecticidal activities, *Paraconiothyrium* sp. SMCD strain (Vujanovic and Germida 2014) was successfully triggered for high spore production (Fig. 6) by allowing depletion of glucose and maintaining fermentation for a 24-h period after glucose depletion. The starvation stress led to spore production, as reported for other fungal endophytes (e.g., Kumar et al. 2011). A similar glucose starvation strategy leads to spore production in *Bacillus subtilis* strains (Monteiro et al. 2005) and could be useful for other spore-producing bacterial endophytes. It is therefore important to understand the microorganism and its growth dynamic to maximize yield and performance (Zhang and Greasham 1999). Furthermore, considerations should be given for formulation approaches to stabilize the microorganism, enable efficient delivery to the host, enhance shelf-life stability and performance (Jones and Burges 1998). At the time of application, the microorganism should typically have a colony forming unit (CFU) count of 10^7^–10^8^ or greater. In our studies with both bacterial and fungal strains, such target CFU has been attained after storage for up to 6 months. The original CFU at the time of harvest was generally 10^9^–10^10^.

After the significant resources spent in a fermentation run, a poor formulation of the product can effectively reduce the value of the product, diminish returns or even fail commercially as a viable product (Bashan et al. 2014; O’Callaghan 2016). Formulation of the product for treatment is, therefore, equally important. The formulation will depend on the intended seed/soil/foliar application method of the product and microorganism type (Pylak et al. 2019). Formulation essentially serves to stabilize and maintain viability and shelf-life of the microorganism of interest until required for application, facilitate its delivery to the target region and ensure its functional attributes at the time of application (O’Callaghan 2016). The formulation strategies and requirements for *Rhizobium* sp. are well-established in the agriculture industry. While many of the plant endophyte formulation developed have been modelled on those rhizobial formulations, the challenge is to integrate consortia within formulations with potential for synergistic effects while maintaining optimum efficiency of the endophytes constituting the consortia (Johnston-Monje and Raizada 2011). This is further compounded when considering consortia consisting of bacterial and fungal strains together. The physiology and growth dynamics of the prokaryotic bacteria and eukaryotic fungi are significantly different and balancing the formulations to enable maintenance of viability, vitality and performance of both entities are important considerations.

## PHA—risk management and mitigation strategies

Process Hazards Analysis (PHA) is a common practice in industrial engineering/manufacturing settings for risk management and mitigation with respect to safety and process undertaking, but not formally used in bioprocessing. In the food processing and beverage industries, approaches such as the Good Manufacturing Practices (GMP), Good Hygiene Practices (GHP), Quality Management Systems (QMS) and Hazard Analysis and Critical Control Points (HACCP) are implemented and followed to guarantee safety of the food and beverage product for consumption (for reviews see Kotsanopoulos and Arvanitoyannis 2017; Panghal et al. 2018). In the fermentation industry elements of the QMS, GMP and HACCP are adhered to and are of critical importance in the pharmaceutical industries. While it may appear that less rigorous systems may be required in the manufacture of plant inoculants, this is not the case if product integrity is to be guaranteed. Therefore, in plant inoculants production, elements of mostly QMS are applied. Although elements of PHA are unconsciously practised in fermentation processes, there is limited information on its implementation and use. Implementation of the PHA approach in scale-up fermentation processes would enhance economic and production success rate of the project by identifying and assessing risks and proposing mitigating solutions.

PHA is the systematic, comprehensive and analytical review of a process based on a set of standards to identify and assess process and operational hazards and their consequences (Nolan 1994). Emergence of PHA strategies and other similar risks assessment approaches arose as a result of recurring and serious incidents involving operators, personnel, machinery, etc., with consequences in loss of productivity, revenue, public trust and overall dependability on meeting demands. Many of the larger companies now have set policies in place for PHAs (Chastain et al. 2017), although with varying approaches to assess risks and address mitigation. Aside from the normal safety procedures in place and Standard Operating Procedures (SOPs), PHA encompasses additional operational safety associated with processes as well as non-safety related process failures which could potentially jeopardize project’s success and result in economic losses. It has to be emphasized that basic operational or general safety procedures are assumed to have been met and in place prior to conducting detailed PHA studies for the specific processes to be started (Cameron et al. 2017). Unless those safety safeguards are in place and properly assessed, a process should never be started. PHA, in the case of a plant endophyte scale-up process, would entail a process analysis at different levels from economic feasibility and financial investments to available technology platform for the process development and production.

As part of the PHA, biosafety and biosecurity risks need to be addressed. The Biosafety Officer oversees and implements general biosafety procedures and enforces the required regulations. However, for every new microorganism which is to be used in a facility a bioprocess review should be conducted by the Biosafety Officer in consultation with the team involved with the project and the new microorganism. Although it is generally perceived that the plant endophytes are naturally occurring beneficial microorganisms that are not harmful to plants, animals or humans, it is important to understand that some of them may be opportunistic pathogens (Abhilash et al. 2016) or producers of toxins. Furthermore, people with immuno-compromised systems might be particularly vulnerable to any kind of microorganisms under certain environmental conditions (Li et al. 2013). It is therefore essential that as much characterization of the bacterial or fungal strain be conducted at the morphological, cultural, biochemical and molecular level to ascertain that the strains in question are not potentially harmful to humans and/or animals. All these characterization details should be provided to the Biosafety Officer by the source laboratory of the microorganism. Furthermore, the dual-use potential of these microorganisms needs to be evaluated and appropriate control measures should be implemented against misuse. Dual-use potential is a scenario when a potentially beneficial research can be used to cause significant harm to public health, the environment, economy or national security (NSABB 2007). This dual-use potential concern is further heightened due to the risk of bioterrorism and calls for a concerted effort by scientists, institutions, government agencies, etc., to raise awareness, educate and regulate research and work with potential for dual-use (Atlas and Dando 2006; Resnik 2010).

The concept of biosafety risk classes, first proposed by the World Health Organization in 1983 is a good baseline to classify microorganisms for laboratory related work and serves as a guiding principle for assuring the safety of humans, animals and the environment to exposure to potential harmful microorganisms (WHO 2004). In an effort to develop a more comprehensive approach to biosafety of plant growth-promoting bacteria, a system of Environmental and Human Safety Index (EHSI) protocol has been proposed (Vílchez et al. 2016), consisting of a battery of tests and evaluation system to accurately assess the safety of bacterial strains. While such systems are essential at the research stage, at the PDO and manufacturing stage the large-scale nature of the process poses risks to personnel and environment, often not considered at the research stage. For example, certain strains of *Penicillium* sp. are known plant endophytes and may not pose a risk at the laboratory stage research. However, at the PDO or scale-up stage due to handling/growing of microorganisms at considerably larger volumes, one has to consider allergenicity to serine proteases (Shen et al. 1999, 2007). For example, the risk of exposure to *Penicillium* sp. to laboratory personnel is likely in a fermentation facility. Plant endophytes pose unknown potential risks to humans due to their relatedness to many known human and animal pathogens (Keswani et al. 2019) and not restrained to a single functional group of microbial species. Therefore, more rigorous characterization at the molecular and metabolomic levels is needed at the research stage and as much detail as possible should be provided prior to scale-up PDO.

## TTP—Technology Transfer Package

Technology Transfer Package (TTP) is a comprehensive document with all the detailed steps and procedures, along with provision for the availability of technical expertise, which would lead a CRO/CMO to conduct a successful fermentation run (Gerson et al. 1998). It is particularly important when a process optimized at a CRO is to transition to a CMO for a manufacturing run. The CMO will reproduce the details in the TTP at the scale similar to that of the CRO before transitioning to manufacturing scale by performing an engineering run, where other minor adjustments may be done prior to proceeding with a manufacturing run. Besides the TTP document, the team or project manager/scientists need to work closely with the CMO team to clarify any concerns the CMO team may have (Gerson et al. 1998). Often the technologists and bioprocess scientists involved in the process development for scale-up are unable to communicate the details of the technology to the recipients due to different perceptions or understanding of the technology (Rogers 2002), especially if the recipients are biologists compared to bioprocess scientists/bioprocess engineers at a CRO/CMO.

The flow of the TTP from a CRO can happen under different scenarios. If the CRO has conducted the PDO for researchers from academia or a start-up, the TTP will be provided to them so that they can contact the CMO of their choice for the scale-up. At their request, the CRO can engage with the CMO to provide clarifications on the TTP or the PDO. In some instances, the PDO may have been conducted by the CMO itself, in which case the TTP will seamlessly flow from the PDO team to the manufacturing team within their internal established systems. This is the ideal situation wherein the PDO and manufacturing occur within the same institution. Many CMOs, however, prefer to focus their resources on meeting manufacturing demands than on PDO and leave the PDO to CROs. The TTP in this transition from a CRO to a CMO therefore becomes very important to the manufacturing process.

Typically included in the TTP are details about the microorganism, shake flask culture media and culture conditions, fermentation media, fermentation parameters, downstream processing details such as harvest, recovery and formulation. Importantly, in-process testing requirements should also be provided. The CRO conducting the scale-up will often make suggestions for additional data which may need to be collected to have a better indication of the performance of the microorganism during fermentation.

## Prospects in fermentation

There is an emerging role of microbial fermentation in building the next-generation bioinoculants. Recent research advancements provide practical tools for improved fermentation of endophytic microorganisms from the fundamental to scaling-up production that enters into a new scientific era together with convex agriculture, food and nutrition domains at the cutting edge of technology. Especially, the emerging next-generation sequencing DNA/RNA techniques merit better integration in genomic and transcriptomic studies for assessing the microbes across variety of fermented systems to elucidate molecular and physiological mechanisms in scale-up production system (Cao et al. 2017). Hence, the current state of scaling-up fermentation required more engineering resources skilled in integrated process development to be engaged from the very beginning in microbial process development and to guide ongoing R&D studies on endophytes, thus ensuring a smooth and profitable path to the large-scale commercial end (Crater and Lievense 2018). In that regards, innovative scientific prospects shift towards autonomous machines and smart engineering systems based on opto-electronic oscillators subjected to self-feedback in analysing a typical nonlinear process (Tatsumura et al. 2021) and set of unknown parameters associated with steps in controlling scale-up production. Further, an integrated process can be optimized assessing an array of different endophytes and media compositions. Additionally, a new OSMAC (One Strain Many Compounds) method was introduced to characterize a plethora of variations among culture conditions (González-Menéndez et al. 2019) that can be largely exploited to discover nutritional requests and endophyte-specific cultivation parameters. While multiple resistant biological contaminants pose challenges for growth of endophytes, the biogenic nanoparticles demonstrate antimicrobial effects and seemed appropriate alternatives to synthetic chemicals and antibiotics (Singh et al. 2018). Further improvements are expected using various chemical epigenetic modifiers, elicitors and precursors, that may be designed to simulate microbial interactions, especially, when coculturing endophytic microorganisms (Venugopalan et al. 2016). Though employing large microbial endophytes datasets combined with comprehensive and deep understanding of the microbial scaling-up may help industries. This includes a complement of molecular gene sequence information starting at the barcoding ITS and 16S rDNA regions and moving to specifically targeted gene sequences information, also involving transcripts and certain enzymes, other analytical chemical data entries including an analysis of metabolomics, volatile chemicals, and microscopy images and of any unique cellular/hyphal/cultural/spore as phenotypical characteristics indicative of the microbial fitness (Orozco-Mosqueda and Santoyo 2021; Strobel 2018; Vujanovic et al. 2019). For researchers, it is vital to design a cutting edge scale-up fermentation platform allowing predictable cultivation and success in microbial bioprocessing, large biomass production, and use of quality bioinoculants for biobased industry’s progress and profits (Rouphael and Colla 2020).

## Conclusions

It is of vital importance for modern agriculture, food, and nutraceutical sectors to continue feeding the expanding world population. To support the ever-growing global population, the bioinoculants, in particular plant endophytes have proved to be a potential strategy to maximize the crop yield in constantly changing environments and thus improving food quality and security. As examples, we presented literature-based and our own expertise on microbial endophytes and scale-up production of prokaryotic *Streptomyces,* and eukaryotic *Paraconiothyrium* and *Penicillium* bioproducts for improved food safety and security, food bioprospecting and biofortification. The challenge in research undertakings has been in scaling-up production of endophytes of potential value as most of them lack the infrastructure or knowhow to transition research discoveries to scale-up production. This review has provided a generalized overview of the fermentation process including pre-, in-, and post-production challenges, the necessity for the implementation of process hazard analysis and the development of technology transfer packages. Innovations are occurring across multiple types of fermentation systems. Scaling-up industrial biotechnology has tremendous potential, while innovation based on large biomass production of bioinoculants remains untapped. The vast diversity of endophytes, their functions and metabolites, coupled with virtually limitless capabilities of next-generation engineering, DNA/RNA and deep learning technologies, translates to immense opportunity for novel alternative bioprocess solutions to emerge from scale-up fermentation-based approaches and manufacturing.

## Data Availability

All data are presented in this paper.

## References

[CR1] Abhilash PC, Dubey RK, Tripathi V, Gupta VK, Singh HB (2016). Plant growth-promoting microorganisms for environmental sustainability. Trends Biotechnol.

[CR2] Atlas RM, Dando M (2006). The dual-use dilemma for the life sciences: perspectives, conundrums, and global solutions. Biosecur Bioterror.

[CR3] Bashan Y, de-Bashan LE, Prabhu SR, Hernandez J-P (2014). Advances in plant growth-promoting bacterial inoculant technology: formulations and practical perspectives (1998–2013). Plant Soil.

[CR4] Behera BK, Varma A (2017). Microbial biomass process technologies and management.

[CR5] Berg G, Rybakova D, Grube M, Köberl M (2015). The plant microbiome explored: implications for experimental botany. J Exp Bot.

[CR6] Berg G, Rybakova D, Fischer D (2020). Microbiome definition re-visited: old concepts and new challenges. Microbiome.

[CR7] Burke F (2008) Practical fermentation technology. In: McNeil B, Harvey LM (eds) Wiley, West Sussex, pp 231–269

[CR8] Cameron I, Mannan S, Nemeth E, Park S, Pasman H, Rogers W, Seligmann B (2017). Process hazard analysis, hazard identification and scenario definition: are the conventional tools sufficient, or should and can we do much better?. Process Saf Environ Prot.

[CR9] Cao Y, Fanning S, Proos S, Jordan K, Srikumar S (2017). A review on the applications of next generation sequencing technologies as applied to food-related microbiome studies. Front Microbiol.

[CR10] Chartrain M, Chu L (2008). Development and production of commercial therapeutic monoclonal antibodies in mammalian cell expression systems: an overview of the current upstream technologies. Curr Pharm Biotechnol.

[CR11] Chastain JW, Delanoy P, Devlin C, Mueller T, Study K (2017). Beyond HAZOP and LOPA: four different company approaches. Process Saf Prog.

[CR12] Chitnis VR, Suryanarayanan TS, Nataraja KN, Prasad SR, Oelmüller R, Shaanker RU (2020). Fungal endophyte-mediated crop improvement: the way ahead. Front Plant Sci.

[CR13] Crater JS, Lievense JC (2018). Scale-up of industrial microbial processes. FEMS Microbiol Lett.

[CR14] Elsallamet MEA, EL-Moslamy SH, El-Al AA, Zahran HF (2021). Scaling-up production of cost-effective and eco-friendly bio-fertilizer and its application on Barley green fodder via IoT hydroponic system. J Genet Eng Biotechnol.

[CR15] Fadiji AE, Babalola OO (2020). Elucidating mechanisms of endophytes used in plant protection and other bioactivities with multifunctional prospects. Front Bioeng Biotechnol.

[CR16] FAO (2011). The state of the world’s land and water resources for food and agriculture—managing systems at risk.

[CR17] FAO (2017). The future of food and agriculture—trends and challenges.

[CR18] FAO (2018). World Food Agriculture—statistical pocketbook 2018.

[CR19] Gerson DF, Himes V, Hopfer R, Khandke L, Kohn F, Komotar A, Krumm P, Machulski J, Weisser A, Sciotto-Brown S (1998). Transfer of processes from development to manufacturing. Drug Inf J.

[CR20] González-Menéndez V, Crespo G, Toro C, Martín J, de Pedro N, Tormo JR, Genilloud O (2019). Extending the metabolite diversity of the endophyte *Dimorphosporicola tragani*. Metabolites.

[CR21] Harvey LM, McNeil B (2008) Practical fermentation technology. In: McNeil B, Harvey LM (eds) Wiley, West Sussex, pp 97–123

[CR22] Hayward A (2008) Practical fermentation technology. In: McNeil B, Harvey LM (eds) Wiley, West Sussex, pp 271–288

[CR23] Johnston-Monje D, Raizada MN (2011). Comprehensive biotechnology.

[CR24] Jones KA, Burges HD (1998) Formulation of microbial biopesticides: beneficial microorganisms, nematodes and seed treatments. In: Burges HD (ed) Springer, Netherlands, pp 7–30

[CR25] Junker BH (2004). Scale-up methodologies for Escherichia coli and yeast fermentation processes. J Biosci Bioeng.

[CR26] Kakes E (2008) Practical fermentation technology. In: McNeil B, Harvey LM (eds) Wiley, West Sussex, pp 289–322

[CR27] Kennedy M, Krouse D (1999). Strategies for improving fermentation medium performance: a review. J Ind Microbiol Biotechnol.

[CR28] Keswani C, Prakash O, Bharti N, Vílchez JI, Sansinenea E, Lally RD, Borriss R, Singh SP, Gupta VK, Fraceto LF, de Lima R, Singh HB (2019). Re-addressing the biosafety issues of plant growth promoting rhizobacteria. Sci Total Environ.

[CR29] Kotsanopoulos KV, Arvanitoyannis IS (2017). The role of auditing, food safety, and food quality standards in the food industry: a review. Compr Rev Food Sci Food Saf.

[CR30] Kumar V, Sahai V, Bisaria VS (2011). High-density spore production of *Piriformospora*
*indica*, a plant growth-promoting endophyte, by optimization of nutritional and cultural parameters. Bioresour Technol.

[CR31] Kumari V, Germida J, Vujanovic V (2018). Legume endosymbionts: drought stress tolerance in second-generation chickpea (*Cicer*
*arietinum*) seeds. J Agron Crop Sci.

[CR32] Li G-X, Wu X-Q, Ye J-R (2013). Biosafety and colonization of *Burkholderia multivorans* WS-FJ9 and its growth-promoting effects on poplars. Appl Microbiol Biotechnol.

[CR33] Mandenius C-F, Brundin A (2008). Bioprocess optimization using design-of-experiments methodology. Biotechnol Prog.

[CR34] Matthews G (2008) Practical fermentation technology. In: McNeil B, Harvey LM (eds) Wiley, West Sussex, pp 3–36

[CR35] Mitter EK, Tosi M, Obregón D, Dunfield KE, Germida JJ (2021). Rethinking crop nutrition in times of modern microbiology: innovative biofertilizer technologies. Front Sustain Food Syst.

[CR36] Monteiro SM, Clemente JJ, Henriques AO, Gomes RJ, Carrondo MJ, Cunha AE (2005). A procedure for high-yield spore production by *Bacillus subtilis*. Biotechnol Prog.

[CR37] Murphy BR, Doohan F, Hodkinson T (2016). A seed dressing combining fungal endophyte spores and fungicides improves seedling survival and early growth in barley and oat. Symbiosis.

[CR38] Murphy BR, Hodkinson TR, Doohan FM (2017). A fungal endophyte consortium counterbalances the negative effects of reduced nitrogen input on the yield of field-grown spring barley. J Agric Sci.

[CR39] Murphy BR, Doohan FM, Hodkinson TR (2018). From concept to commerce: developing a successful fungal endophyte inoculant for agricultural crops. J Fungi.

[CR40] Nicolaidou V, Nicolaou P, Nicolaou SA (2019). Transforming a cookbook undergraduate microbiology laboratory to inquiry based using a semester-long PBL case study. Adv Physiol Educ.

[CR41] Nolan DP (1994). Application of HAZOP and what-if safety reviews to the petroleum, petrochemical and chemical industries.

[CR42] NSABB (2007). Proposed framework for the oversight of dual use life sciences research: strategies for minimizing the potential misuse of research information.

[CR43] O’Callaghan M (2016). Microbial inoculation of seed for improved crop performance: issues and opportunities. Appl Microbiol Biotechnol.

[CR44] Orozco-Mosqueda MdC, Santoyo G (2021). Plant-microbial endophytes interactions: scrutinizing their beneficial mechanisms from genomic explorations. Curr Plant Biol.

[CR45] Panghal A, Chhikara N, Sindhu N, Jaglan S (2018). Role of Food Safety Management Systems in safe food production: a review. J Food Saf.

[CR46] Parnell JJ, Berka R, Young HA, Sturino JM, Kang Y, Barnhart DM, DiLeo MV (2016). From the lab to the farm: an industrial perspective of plant beneficial microorganisms. Front Plant Sci.

[CR47] Pham JV, Yilma MA, Feliz A, Majid MT, Maffetone N, Walker JR, Kim E, Cho HJ, Reynolds JM, Song MC, Park SR, Yoon YJ (2019). A review of the microbial production of bioactive natural products and biologics. Front Microbiol.

[CR48] Pylak M, Oszust K, Frąc M (2019). Review report on the role of bioproducts, biopreparations, biostimulants and microbial inoculants in organic production of fruit. Rev Environ Sci Biotechnol.

[CR49] Rana KL, Kour D, Kaur T, Devi R, Yadav AY, Yadav Y, Dwaliwal SH, Saxena AK (2020). Endophytic microbes: biodiversity, plant growth-promoting mechanisms and potential applications for agricultural sustainability. Antonie Van Leeuwenhoek.

[CR50] Reinhold-Hurek B, Hurek T (2011). Living inside plants: bacterial endophytes. Curr Opin Plant Biol.

[CR51] Reisman H (1988). Economic analysis of fermentation processes.

[CR52] Resnik DB (2010) Can scientists regulate the publication of dual use research? 10.2202/1941-6008.1124 PMC3134283 21760964

[CR53] Robl D, da Silva Delabona P, Mergel CM, Rojas JD (2020). The capability of endophytic fungi for production of hemicellulases and related enzymes. BMC Biotechnol.

[CR54] Rogers EM (2002). The nature of technology transfer. Sci Commun.

[CR55] Rouphael Y, Colla G (2020). Toward a sustainable agriculture through plant biostimulants: from experimental data to practical applications. Agronomy.

[CR56] Running JA, Bansal K (2016). Oxygen transfer rates in shaken culture vessels from Fernbach flasks to microtiter plates. Biotechnol Bioeng.

[CR57] Santos MS, Nogueira MA, Hungria M (2019). Microbial inoculants: reviewing the past, discussing the present and previewing an outstanding future for the use of beneficial bacteria in agriculture. AMB Expr.

[CR58] Shen HD, Lin WL, Tam MF, Wang SR, Tzean SS, Huang MH, Han SH (1999). Characterization of allergens from *Penicillium oxalicum* and *P. notatum* by immunoblotting and N-terminal amino acid sequence analysis. Clin Exp Allergy.

[CR59] Shen H-D, Tam MF, Tang R-B, Chou H (2007). Aspergillus and Penicillium allergens: focus on proteases. Curr Allergy Asthma Rep.

[CR60] Singh V, Haque S, Niwas R, Srivastava A, Pasupuleti M, Tripathi CKM (2017). Strategies for fermentation medium optimization: an in-depth review. Front Microbiol.

[CR61] Singh P, Garg A, Pandit S, Mokkapati VRSS, Mijakovic I (2018). Antimicrobial effects of biogenic nanoparticles. Nanomaterials (basel, Switzerland).

[CR62] Soccol CR, Costa ESFd, Letti LAJ, Karp SG, Woiciechowski AL, Vandenberghe LPdS (2017). Recent developments and innovations in solid state fermentation. Biotechnol Res Innov.

[CR63] Stanbury PF, Whitaker A, Hall SJ (1995) Principles of fermentation technology (2nd edn). In: Stanbury PF, Whitaker A, Hall SJ (eds) Pergamon, Amsterdam, pp 35–91

[CR64] Strobel G (2018). The emergence of endophytic microbes and their biological promise. J Fungi (basel).

[CR65] Takahashi M, Aoyagi H (2018). Practices of shake-flask culture and advances in monitoring CO2 and O2. Appl Microbiol Biotechnol.

[CR66] Tatsumura K, Yamasaki M, Goto H (2021). Scaling out Ising machines using a multi-chip architecture for simulated bifurcation. Nat Electron.

[CR67] Turner TR, James EK, Poole PS (2013). The plant microbiome. Genome Biol.

[CR68] Ude C, Hentrop T, Lindner P, Lücking TH, Scheper T, Beutel S (2015). New perspectives in shake flask pH control using a 3D-printed control unit based on pH online measurement. Sens Actuators B Chem.

[CR69] Venugopalan A, Potunuru UR, Dixit M, Srivastava S (2016). Effect of fermentation parameters, elicitors and precursors on camptothecin production from the endophyte *Fusarium*
*solani*. Bioresour Technol.

[CR70] Vílchez JI, Navas A, González-López J, Arcos SC, Manzanera M (2016). Biosafety test for plant growth-promoting bacteria: proposed environmental and human safety index (EHSI) protocol. Front Microbiol.

[CR71] Vujanovic V, Germida JJ (2014) Endophytic microbial symbionts in plant prenatal care. In: Organization, W.I.P. (ed) Canada, p 154

[CR72] Vujanovic V, Germida JJ (2017). Seed endosymbiosis: a vital relationship in providing prenatal care to plants. Can J Plant Sci.

[CR73] Vujanovic V, Kim SH, Lahlali R, Karunakaran C (2019). Spectroscopy and SEM imaging reveal endosymbiont-dependent components changes in germinating kernel through direct and indirect coleorhiza-fungus interactions under stress. Sci Rep.

[CR74] Weuster-Botz D, Altenbach-Rehm J, Arnold M (2001). Parallel substrate feeding and pH-control in shaking-flasks. Biochem Eng J.

[CR75] WHO (2004). Laboratory biosafety manual.

[CR76] Willaert RG (2021). Yeast biotechnology 4.0. Fermentation.

[CR77] Zhang J, Greasham R (1999). Chemically defined media for commercial fermentations. Appl Microbiol Biotechnol.

